# Technology and Information Sharing in Disaster Relief

**DOI:** 10.1371/journal.pone.0161783

**Published:** 2016-09-01

**Authors:** Benedikte Bjerge, Nathan Clark, Peter Fisker, Emmanuel Raju

**Affiliations:** 1 Faculty of Science, University of Copenhagen, Copenhagen, Denmark; 2 Faculty of Law, University of Copenhagen, Copenhagen, Denmark; University of Texas at San Antonio, UNITED STATES

## Abstract

This paper seeks to examine the extent to which technological advances can enhance inter-organizational information sharing in disaster relief. Our case is the Virtual OSOCC (On-Site Operations Coordination Centre) which is a part of the Global Disaster Alert and Coordination System (GDACS) under the United Nations Office for Coordination of Humanitarian Affairs (UN OCHA). The online platform, which has been developing for more than a decade, provides a unique insight into coordination behaviour among disaster management agencies and individual actors. We build our study on the analysis of a complete database of user interaction including more than 20,000 users and 11,000 comments spread across approximately 300 disaster events. Controlling for types and severities of the events, location-specific vulnerabilities, and the overall trends, we find that the introduction of new features have led to increases in user activity. We supplement the data-driven approach with evidence from semi-structured interviews with administrators and key users, as well as a survey among all users specifically designed to capture and assess the elements highlighted by both interviews and data analysis.

## Introduction

The exchange of relevant information is critical in the immediate aftermath of a major disaster. Within the first 72 hours, stakeholders work against the clock to find and rescue survivors, provide life-saving medical treatments, and set up the infrastructure for a long-term humanitarian intervention. However, the disorder of inter-organizational information sharing in this period often leads to overlapping initiatives and the extensive mismanagement of resources, which is in turn linked to the loss of lives and livelihoods on the ground. This is clear from reports following the 2004 Indian Ocean earthquake and tsunami, Hurricane Katrina, and the 2010 Haiti Earthquake ([1]; [2]; [3]).

Fortunately, the advances and access to new technologies have helped progress information sharing efforts in the field. Information communication technologies (ICTs) in particular are changing the way stakeholders communicate and share data within and across borders during crises. Web portals such as ReliefWeb and HumanitarianResponse.info continue to provide up-to-date status reports about ongoing emergencies. At the same time, open source web tools and data deposits are emerging alongside social and technical networks to facilitate more timely and efficient methods for collecting and processing data. In the aftermath of the 2010 Haiti earthquake, members of the affected community and global volunteers used mobile applications and social media networks, such as OpenStreetMaps and Twitter, to coordinate data and information with disaster responders [3]. These collaborations were largely effective, and also worked to bring wider attention to the practices of crowdsourcing, crisis mapping and big data analytics to the traditional relief and response communities [4].

As these technologies continue to advance, we should expect to see an increase in the efficiency of information exchange as well as the amount of information shared among relevant stakeholders in disaster relief networks. This paper aims to assess these effects through an extensive case study of the UN OCHA hosted Virtual On-Site Operations Coordination Centre (Virtual OSOCC). With more than 20,000 registered users within the disaster response community, over the last 15 years, the system has evolved technically and operationally to provide stakeholders with various levels of information in the immediate relief phase following a disaster event. From situational overviews and maps, to the requests and status for relief teams and items, it has become a central information portal for global relief actors. This paper examines the impact of specific changes to the technological architecture and functionality of the Virtual OSOCC on user activity and the amount of information shared over time. We first employ meticulous quantitative methods on a unique data set of complete user interaction and show that the introduction of new features that enhance ease-of-use and usefulness greatly increases the amount of information shared in the system. For instance, putting in place features that help with the organization of information about relief teams and maps have led the number of pieces of information of those types to be tripled and quadrupled respectively. We then support these findings by applying a modified Technology Acceptance Model to data collected through semi-structured interviews and a survey distributed to all active users, which provide useful insights into the perceptions and opinions of users as to the effectiveness of changes made to the system.

## Information Sharing in Disaster Relief Coordination

Information sharing within the field of disaster relief can be defined within the broader context of coordination. Coordination continues to be a complicated phenomenon, which has different meanings to different stakeholders ([5]; [6]; [7]; [8]; [9]). From the agendas of policymakers, to the operational needs of international and domestic relief entities, the concept is ontologically reshaped by the varying goals and activities of a wide range of actors. For the purposes of this paper, we assent with Malone and Crowstone, who define coordination as “the act of managing interdependencies between activities performed to achieve a goal” [10]. This definition provides that there are various interdependencies between organizations involved in disaster relief and all responding organizations working towards a common goal of saving lives.

Within this framework, we can further identify a number of activities which support coordination, such as communication, collaboration, and cooperation [9]. Information sharing falls within the domain of communication, and is also recognized by the IFRC as one of three primary levels of coordination [11] (the other two being collaboration and joint-strategic planning). While all of these activities are in some way interconnected and equally relevant when discussing coordination, it is important to recognize that at the level of information sharing, the exchange of information does not necessarily require or involve any other form of action (see for example; Saab, Maldonado, Orendovici, Tchouakeu, van Gorp, Zhao 2008; Altay and Labonte 2014). In other words, the action of information sharing can be isolated and analyzed separate from its impact on coordination efforts as a whole (ibid). Indeed, that is the approach taken in this paper. However, to fully understand the implications of information sharing in coordination it is also useful to understand the issues surrounding these interdependencies, and the ways in which technology works to support and/or complicate developments in this area.

### Barriers to Information Sharing in the Age of Technology

The organized exchange of information in the disaster relief sector has become more complex than ever. Over the past three decades, the swell of aid actors has done little to change the *ad hoc* ways in which relief organizations coordinate and exchange resources and information on a case-by-case basis [12]. Often, there is more information than necessary and sometimes not the relevant information that a particular stakeholder requires. This process of information sharing and exchange needs to be channelized and coordinated. The challenge therefore *… is creating an information infrastructure that is sufficiently flexible to manage the dynamic exchange of information among the participating entities in an inter-organizational system, but sufficiently ordered to ensure that the relevant information gets to the responsible parties in valid format and in time to support effective action* [13]. One method for achieving this is through the effective use of ICT platforms. ICTs have become a primary asset among stakeholders for the coordination and sharing of information in all phases of the disaster management cycle ([14], [15]). From two-way radios and mobile phones, to humanitarian web forums and social media sites, these platforms function as essential conduits for the timely exchange of information in disaster relief efforts.

However, this progression has also resulted in new and constantly changing environments, where diverse groups of actors struggle to function in a coherent manner. The radical increase in stakeholders, along with the emergence of new technical platforms and tools, have created competitive and parallel initiatives, which works against establishing synchronized efforts for information sharing [16]. NGOs, local governments, international institutions, and private entities, each with their own systems and methods for communicating and distributing information, tend to scatter knowledge and create confusion. For instance, even within the disaster management community stakeholders are not always able to distinguish among information channels, such as Reliefweb, Preventionweb, and the UN OCHA Virtual OSOCC. This is problematic because in the first 72 hours following a disaster stakeholders may lose valuable time if they are uncertain where to find—or must look at multiple sources to gather—the relevant information. Additionally, there are major social, legal and ethical issues in information exchange during disasters. This can be seen in patterns of interaction preferred by emergency managers [17] whether they preferred to exchange information using emails and meetings (as a way of having personal contact) or use analytical decision support systems. It is also worth noting the huge digital divide across the world and is certainly important to information exchange in disasters [18]. Further, while in current times there are large amounts of data freely available online [19], it does not become useful unless this data is processed as organized information. In this regard, it is undeniable that not all information goes through a rigorous ethical test during disasters. Other concerns, such as privacy and legal issues around data sharing can also hinder the effective exchange of relevant data in disaster scenarios. Therefore, it is crucial to state that information processing and exchange must adhere to social, ethical and legal norms. While these issues are certainly important to address, in the context of this paper, what is most important is that, there is an absence of understanding into the usability and effectiveness of existing platforms. As these systems evolve, the changes may have important implications for end-users. Their ability to find, process, and share information is directly linked to their understanding and familiarity with the technology. Therefore, this paper asserts that in order to better integrate new technologies and stakeholders into the picture, it is first relevant to assess the effectiveness and limitations of existing platforms from the perspectives of the users.

### Technology Acceptance Model

On a broader level this paper relates to how and why individuals accept and use technological platforms. The implementation of these instruments (e.g. computer systems, software, email) does not alone ensure the adoption and use of the technology by end-users. Factors such as the design and changes to the technology, as well as the user’s experience, needs, and perceptions, all contribute to the overall use (and performance) of the system. A common method for conceptualizing the causal linkages among these variables is through the application of a Technology Acceptance Model (TAM) ([20]; [21]). The TAM was introduced in the 1980s, and works as a bridge between social psychology with information systems theories for the study of technology adoption by users. In its most basic form, the model suggests that the behavioural intent of users, and the actual system usage, are dependent on both external variables (e.g. system features, time, and experience) and the perceived usefulness and ease-of-use of the technology by the users [20]. For instance, individuals might reject a new web-based system for sharing information if they have difficulty understanding and using the system, or don’t recognize the benefits in doing so. Conversely, if the users perceive the system as easy to use and performance enhancing, their acceptance and use of the system will increase.

**Fig 1:**
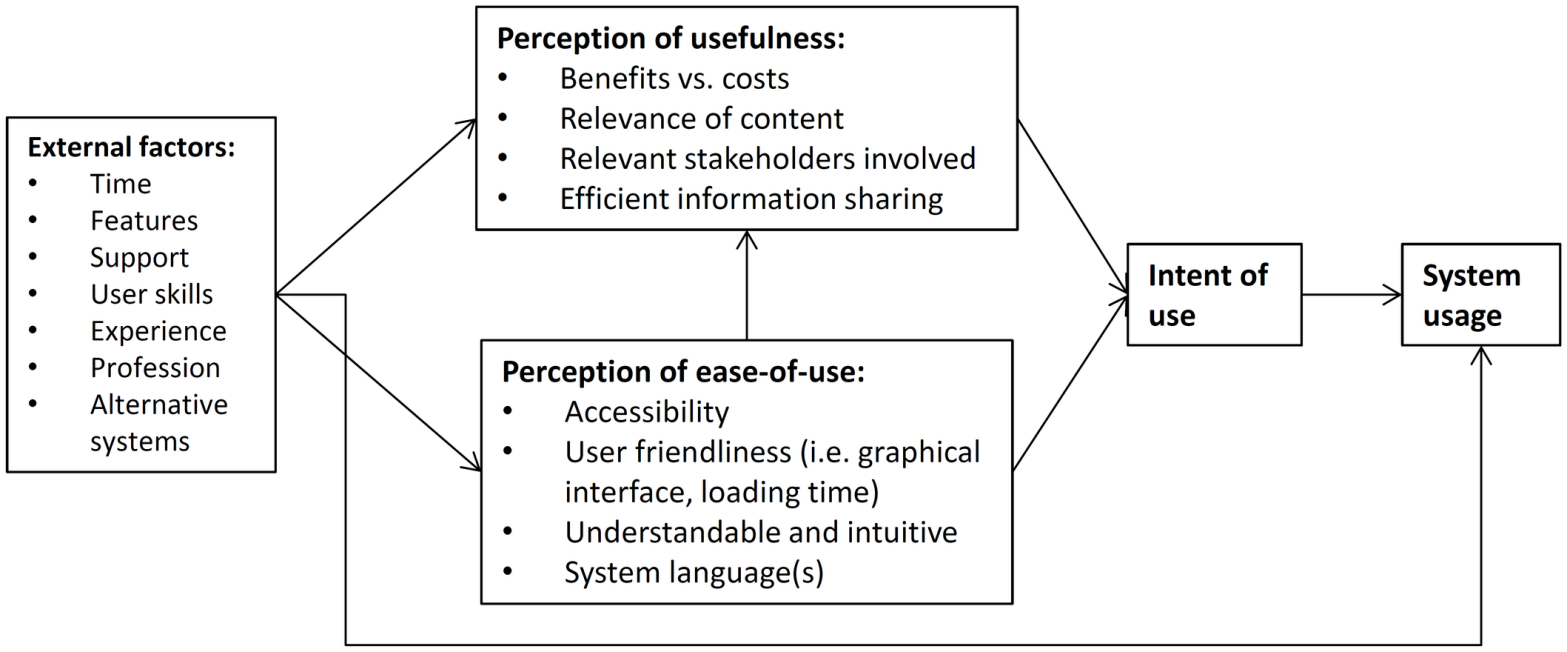
Technology Acceptance Model.

Since its inception, the TAM has been extended and applied in various dimensions to provide researchers with more predictive methods for understanding and mapping user interactions with technology ([20]; [21]; [22]). For this paper, we have adopted a modified version of the TAM framework to guide our assessment of how changes made to the Virtual OSOCC system (and other external factors) may impact information sharing and coordination among users (see Fig 1). We correlate positive system acceptance and usage to an increase in the amount of shared information, along with the satisfaction of users with the system performance. While controlling for various factors, we will quantitatively test direct causality between external factors and system use, and validate our results with survey and interview data which reflect how the same variables impact the perceptions and behavioural attitudes of users towards the system. External factors include feature changes in the Virtual OSOCC, time, user experience and professional backgrounds, and alternative systems. We define perceived ease of use by whether users find it easier to access, use, and understand the system based on changes to the aforementioned variables. Finally, we define perceived usefulness as the extent to which users believe that the changes have resulted in more efficient information sharing among registered users.

## The Virtual OSOCC

A growing need within the international disaster relief community to standardize the coordination of information and efforts among urban search and rescue (USAR) teams after earthquakes prompted the establishment of the International Search and Rescue Advisory Group (INSARAG) in 1991. Within the Emergency Service Branch (ESB) of the United Nations Office of Humanitarian Affairs (UN OCHA), INSARAG developed the concept for an On-Site Operations Coordination Centre (OSOCC) aimed at improving the coordination between local Governments, USAR teams, and other international responders following a disaster. As a ground level mechanism, the OSOCC was an effective initiative. Apart from ReliefWeb, which was launched in 1996 by UN OCHA and acted as a broader, open information sharing tool for all humanitarian networks, a technological platform for addressing specific coordination needs within disaster management sector did not yet exist. As a result, ESB began planning for an affordable, easy to manage solution that could provide real-time information sharing across the globe in the early phases of disasters.

The first version of the Virtual OSOCC was developed and launched between 1999 and 2001. Sharing the ReliefWeb server, the system architecture was supported by classic Microsoft applications. The web portal was managed through Active Server Pages (ASP) and ActiveX controls, and data was stored in an Access database on the backend. The technical and operational management of the system was maintained by the system architect and a handful of UN employees and interns, who shuffled these responsibilities in among their other tasks. Because there was no mandate or select budget to operate the system, it was designed to be highly user driven and relied on the voluntary input from stakeholders in the first phases of a disaster. In this context, the initial system primarily functioned as an interactive discussion blog for emergencies, meetings, and training among registered, relevant actors from within the UN and USAR communities.

In 2003, a second version of the system was launched through the migration to servers at the UN Offices in Geneva. The migration was necessary as the number of users and need for better processing power within the system began to grow. At the same time, to assist with the management of data and the administration of the web pages, the system architecture was upgraded to a .NET framework with SqlServer 2005. Over the next 12 years the architecture would remain fairly unchanged, with the exception of a migration to SqlServer 2008 and Windows 2012 Webserver in 2015. However, in that timespan there was an increased focus on user behavior and feedback which resulted in various changes to the system features in terms of functionality and usability. The most relevant of these changes included: The integration of the system with the GDACS (Global Disaster Alert and Coordination System) platform, and the implementation of a GLIDE (GLobal IDEntifier) number for all new disaster events, and map feeds (2004); a feature for uploading file attachments with user comments (2005); on-line UNDAC (United Nations Disaster Assessment and Coordination) alerts, a registration feature for relief teams and relief items; and a separate simulator section to support training and exercises (2006); mandatory acceptance of Terms of Service for all users and the integration of a Hazard Identification Tool (HIT) (2010); a standardized structure for discussions and the introduction of on-line user surveys (2011); a multilingual interface of static content and ongoing improvements to the discussion structure based on feedback from the working groups within the system (2014-Present).

Of the changes made to the system over the years, this paper is particularly interested in examining changes in user behavior after the redesign of the systems architecture in 2003, and the integration of the GDACS, maps, attachments, relief teams, and relief items features. The new features were introduced to make it easier for users to interact and share information within the system. For instance, the GDACS alert system sends out text message alerts to all system users when an event occurs. This allows them to closely follow the latest news and situation reports in order to decide within hours how to react. The ability to upload and share maps and other attachments provides all relevant users with centralized access to the same information, such as impact trajectories, damage assessments, and situational overviews. Along similar lines, the introduction of the relief teams and items features give users the opportunity to add, update, and monitor the deployment status of teams and items for each event. Through a standard template, users register general details about the teams and items, and select the appropriate status from a dropdown menu. Status options include monitoring, mobilising, deployed and stand-down for teams, and considered, dispatching, delivered and canceled for items.

The current system design is the result of many years of interaction between the administrators and users, where new features are introduced when relevant, and altered or removed if they prove to be unnecessary. In fact, it is this flexible dynamic which has worked to solidify the reputation and usefulness of the system among the disaster management community. Despite being a closed system only accessible to disaster managers, the Virtual OSOCC has attracted more than 20,000 users throughout its lifetime, and this number continues to increase annually by 20 percent. As the leading technical platform for international disaster relief over the last two decades, our analysis of the Virtual OSOCC contributes to a more definitive understanding of the implications of technological advances for stakeholders in the relief sector. The following section introduces the method and results from our analysis of the system.

## Methodology

The methodology of the study relies on a combination of quantitative and qualitative approaches. We combine a complete activity-log from the Virtual OSOCC database covering the entire lifetime of the system with semi-structured interviews as well as a survey administered to active users. These different data sources enable us to assess how exogenous technological changes to the Virtual OSOCC affect user behavior. To our knowledge, this constitutes the first attempt to systematically study the Virtual OSOCC and to test the importance of technical improvements for inter-organizational information sharing using longitudinal data.

### Data collection

Our main statistical analysis is based on a complete log of user activity extracted from the Virtual OSOCC server. The data contain information about all users and their corresponding activity in the system for each disaster event created in the Virtual OSOCC since the launch in 2001 up until (including) the Nepal Earthquake on April 25, 2015. In principle all users can create a disaster event; however the vast majority of events created by administrators in response to calls for international assistance.

The database includes three layers of information. First, disaster level data includes information about the type of the disaster, the countries hit, the time and date of the creation of the disaster event, the timespan in which the event was active, the number of unique users that actively contributed information regarding the event, the total number of comments made, the number of maps, attachments, relief teams and items, as well as the number of moderators moderating the content of the page. The final sample consists of 215 disaster events. Out of these, earthquakes, floods, and storms constitute the most frequent disaster types recorded with 34, 29 and 26 percent of the events respectively.

The second data level includes general information about users including gender, the name of the organization or institution they represent, account creation time, the last time they logged on to the system, and average user statistics such as the total number of comments made and the number of relief teams and items added. We aggregate the user level information by disaster in order to combine it with the disaster-level data.

The third layer of data includes all ca. 11,000 comments created by the users throughout the lifetime of the system. For each disaster we observe all actual comments made by each user as well as the time the comment was created. We categorize user comments in order to isolate the effect of software improvements on information sharing specifically. The list of categories is provided in the Supporting Information S1 Table. The comment level data was then merged by category with feature-level data and aggregated to the disaster level allowing us to study the effects on the actual amount of information shared (whether included in a comment or added through a feature) of introducing system features that simplify and sort a certain type of information. Thus the resulting unit of analysis in the quantitative analysis is a disaster.

**Fig 2:**
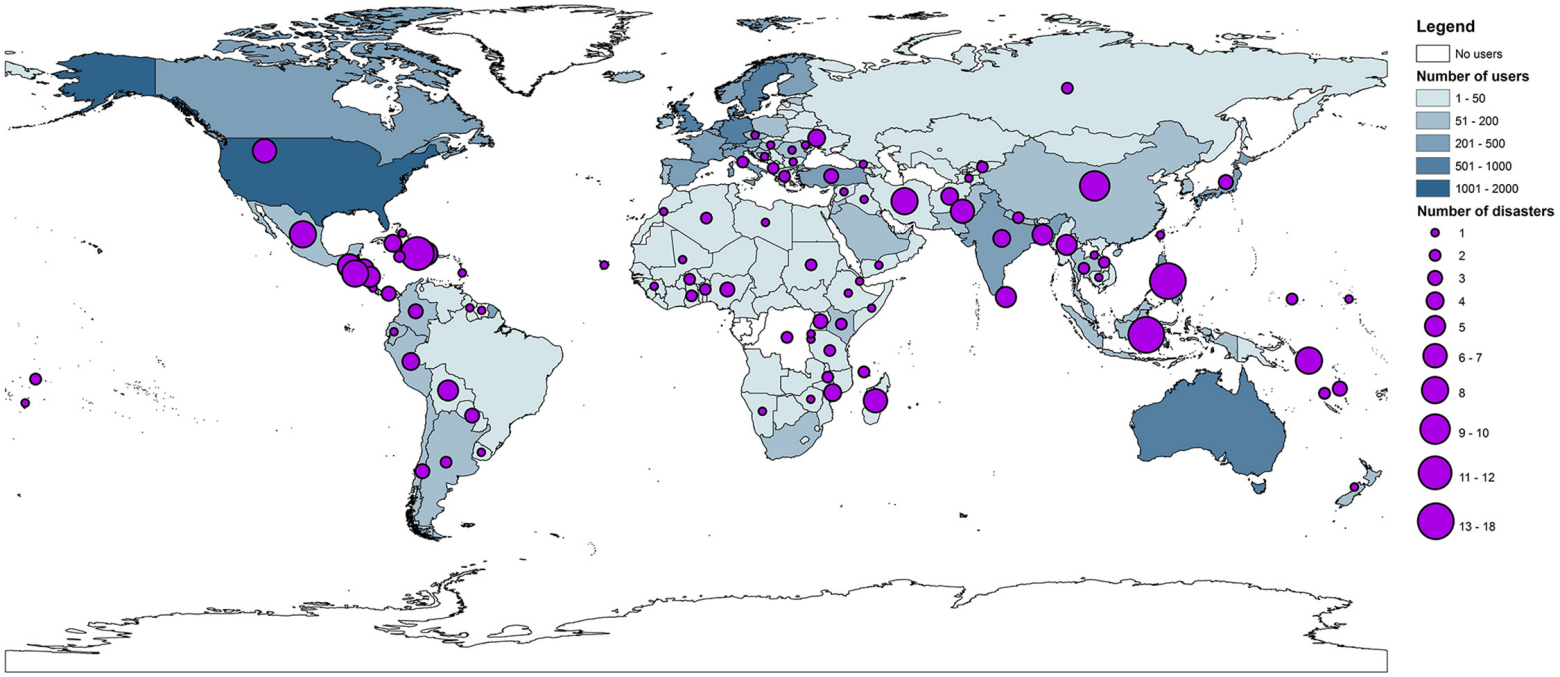
Number of users and number of events in the Virtual OSOCC. Country colors indicate the number of users; dot size indicates the number of disaster events by country. Country outlines downloaded from naturalearthdata.com.

Fig 2 shows the global distribution of events and users. Users of the Virtual OSOCC primarily come from western countries with the United States (1,756), Germany (917), and Australia (650) in the top three. The Asia-Pacific region and Latin America are the regions hardest hit with 44 and 28 percent of all recorded disasters, respectively. At the country-level, Philippines (18), Indonesia (15), and Haiti (12) have experienced the largest number of recorded disaster events in the Virtual OSOCC.

In order to complement the quantitative data and assist with the design and validation of the user survey, we piloted a series of semi-structured interviews with Virtual OSOCC administrators and later a random sample of users. The aim of interviewing first the administrators was to learn from their experiences with the system and establish a baseline for expectations with regards to user behavior. These interviews included a selection of individuals in managerial and operational roles from the GDACS Secretariat and service branches. The administrative interviews took place between February and June 2015 in person and over the phone. User interviews were carried out over the phone or Skype throughout June 2015. Eight individuals were randomly drawn from the entire population of system users. Because we wanted to gauge the experience of the users over the lifetime of the system, we controlled the sample for users that had been registered prior to 2004 and that were currently active (i.e. had logged in and contributed comments to the Virtual OSOCC within the last year). The interview guide reflected on the history of the users and the Virtual OSOCC; user friendliness of the Virtual OSOCC; the purposes and overall performance of the system; different actors using the Virtual OSOCC; experiences particularly related to changes in the system and on the theme of information sharing. These interviews were transcribed and data analyzed by categorizing according to the questions asked. The important issues highlighted in the interviews are presented in the later parts of the paper.

As a further supplement to our quantitative findings, we conducted an online survey among the group of users active during the last 5 years. This group consists of around 5,000 users out of whom 1,044 (21 percent) completed the survey. An important aspect of the survey design is that this was done after analyzing the quantitative user data and the semi-structured interviews to ensure consistency and increase relevance between the different data collection methods. For instance, the user survey includes a section on each of the main changes to the Virtual OSOCC with questions regarding the specific change and how this change affected the ease-of-use and usefulness of the system. These questions were formulated as statements following the Likert scale approach to ordered responses.

Descriptive statistics regarding the characteristics of the users surveyed are presented in Table 1. The users are predominantly men: 85 percent of the respondents are male and the median respondent is 43 years old. As expected, the descriptive statistics paint a picture typical of active users. Only 4 percent of the users surveyed state that they never access the Virtual OSOCC, while two-thirds access the system both during and between disaster events. Out of these, some 47 and 45 percent visit the site daily or hourly within the first week of the disaster, respectively. Regarding the type of contributions made to the system by users, the largest share of users have contributed to the system by using the relief team feature, while the second and third most important type of contribution are user comments and situation reports.

**Table 1:** Descriptive statistics: User survey.

	Mean	Std. Dev.
Gender (male = 1)	0.852	0.356
Age (years)	43.198	9.425
Registered to receive GDACS alerts	0.672	0.47
What type of information have you contributed to the VO?		
Attachments	0.297	0.457
Comments	0.392	0.488
Relief teams	0.441	0.497
Relief items	0.169	0.375
Maps	0.201	0.401
Situation reports	0.332	0.471

Observations: 1,044

In the next section we outline our strategy of exploiting the user data taken from the activity-log of the Virtual OSOCC to assess how external factors influence user behavior. We then go on to present first the quantitative and then the qualitative results before concluding.

### Estimation strategy

In order to test quantitatively the effect of external factors on user behavior, the optimal scenario is to compare actual and counterfactual outcomes (that is, what would have happened to user behavior in the absence of the updates to the system). In other words, one would ideally like to compare how the same users would have behaved with and without the changes to the technology. However, this is not possible since at a given point in time a user cannot have two simultaneous existences. The next best alternative is to compare outcomes of the users exposed to the change to those of a comparison group of unexposed users. Yet, since there exists no comparable technology to the Virtual OSOCC and it is not possible to limit a system intervention to a subset of the users, this approach is not viable. Rather we compare ex post behavior for users with data on their behavior before the system intervention. This approach is also referred to as the reflexive method of impact, where the users’ behavior before the intervention functions as a comparison or control outcome. This method is particularly relevant for the present study as the entire population of users is affected by the system interventions, in which case there is no scope for an external control group. Moreover, since the behavior of the users is observed over the entire life period of the system, structural changes in behavior can be tested for [23].

The main threat to the reflexive method is the presence of other time-varying (external) factors. For instance, imagine that right after the introduction of the new relief teams feature, users of the Virtual OSOCC start announcing a larger share of the actual number of teams deployed in the system. Although this increase in the number of relief teams announced may be due to the system change (through awareness of the usefulness of reporting team deployment), it may also be because the first events following the system change take place in countries considered to be particularly vulnerable or because certain types of disasters tend to generate more activity. To distinguish these effects it is therefore important to account for other factors explaining user behavior. The factors that are likely to affect information sharing can largely be grouped into either disaster specific characteristics or characteristics of the country hit by the disaster.

Disaster specific characteristics include the type and severity of the disaster. We account for disaster type as the nature of assistance needed in the immediate aftermath of the disaster depends heavily on the type of the disaster. For instance, earthquakes in urban areas normally require more search and rescue teams compared to a flood. Additionally, more severe disasters are likely to spark more activity in the Virtual OSOCC as the need for assistance generally increases with the severity of the disaster. To account for the severity of disasters we combine the Virtual OSOCC activity log with data from the Emergency Events Database (EM-DAT) on the number of deaths caused by each disaster. A number of recoded disaster events in the Virtual OSOCC are not defined as a disaster in the EM-DAT database. For a disaster to be entered into the EM-DAT database at least one of the following criteria must be fulfilled: (i) Ten or more people reported killed, (ii) Hundred or more people reported affected, (iii) Declaration of a state of emergency, (iv) call for international assistance. For these cases (20 observations), we complement the information from the database with other data sources, often summarized by Wikipedia. On average, the number of deaths in a disaster event in our sample is 4,406 people.

Country specific characteristics include the vulnerability of affected countries and their level of democratization. The vulnerability of the affected country is likely to determine the involvement of the international community and thereby the level and type of activity observed in the Virtual OSOCC. We measure a country’s vulnerability by their Gross National Income (GNI) per capita using the World Bank Indicators. To ensure comparability across countries and years, GNI per capita is converted to a common currency at purchasing power parity. The mean GNI per capita of affected countries is 5,790—well below the global average of about 13,000 in 2010. We further account for the level of democratization since relief actions in some cases has been hindered by the lack of approval by the affected country’s government to enter the country in relation to rescue operations or distribution of water, food and other necessities; for instance during the Cyclone Nargis in Burma in 2008 where the military regime denied workers access to affected areas. The level of democratization is measured using the Polity IV Annual Time-Series database. The data categorize democratic and autocratic patterns of authority and regime changes in all independent countries with total population greater than 500,000 in 2014. For countries smaller than 500,000 inhabitants we use information collected from other sources to categorize the level of democracy. For disasters that hit more than one country we take the population weighted average of the affected countries GNI per capita and democratization index, respectively.

The regression that we seek to estimate by OLS in order to evaluate the impact from external factors on user behavior in the Virtual OSOCC can be written as follows:

$$\begin{array}{c} y_{jt}=\alpha +\theta change_{t}+\beta_{1}D_{jt}+\beta_{2}X_{it}+\gamma_{t}+\delta +\mu_{jit} \end{array}$$ (1)

where *y*_*jt*_ is a vector of outcome measures for disaster j at time t, and the variable *change*_*t*_ is an indicator variable for the change made to the Virtual OSOCC. As previously mentioned, the changes we consider in our baseline specification are: i) Re-design (2003), ii) introduction of GDACS alerts (2004), and iii) the introduction of four features: relief teams (2006), relief items (2006), maps (2004), and attachments (2005). The vectors *D*_*jt*_ and *X*_*it*_ contain disaster and country specific characteristics, respectively. We also include region specific fixed effects (*δ*) as well as time fixed effects (*γ*_*t*_). We define the regions based on the United Nations’ five geopolitical regional groups: the African Group, the Asian-Pacific Group, the Eastern European Group, the Latin American and Caribbean Group, and finally the Western European and Others Group. Time fixed effects are included in order to account for increasing user activity in the Virtual OSOCC over time. Finally, *μ*_*jit*_ is the error term containing all unobserved characteristics.

We consider nine outcome measures that all approximate different aspects of user activity in the Virtual OSOCC. These outcomes can broadly be categorized into three groups: outcomes related to the overall use of the Virtual OSOCC, outcomes related to user behavior, and outcomes related to specific types of information. The first group of outcomes includes (i) the unique number of active user counts by recorded disaster, and (ii) the number of different organizations/institutions active by disaster. These outcomes will allow us to investigate how the number and type of users are related to the type and severity of the disaster, the vulnerability of the country hit and the type of user activity in the system. The user related outcomes include (iii) the number of comments added during the first day following an event creation, (iv) the total number of comments created by disaster, and (v) the number of user comments by comment type and disaster. The third group of outcomes relate to specific pieces of information. These are contained in either comments or the different system features introduced in the system. For instance, we investigate whether the number of comments related to relief teams increased after the relief team feature was introduced. Specifically, the third group of outcomes includes the number of user-specific comments about (vi) relief teams, (vii) relief items, (viii) maps, and (viiii) attachments. We finally go even more into detail with subcategories of the relief teams feature.

## Quantitative Results

Based on a modified version of the TAM framework, we quantitatively test how user behavior related to information sharing changes with technological updates linked to external factors outside the control of the individual user. For this, we start by estimating Eq (1) using the Virtual OSOCC activity log. Results are presented in Table 2. Four dependent variables (*y*_*jt*_) are considered: unique user count, number of users active in disaster event, total number of comments, and the number of comments on the day the disaster event was created. We find that the system re-design in 2003 positively affect the number of users that commented in the system within the first 24 hours after the disaster event was activated in the Virtual OCOSS (column 1). Surprisingly, there is no detectable increase in the number of users that respond within a day compared to before the GDACS alerts system was introduced. This should be seen in relation to our user survey where 67 percent state that they are registered to receive GDACS alerts, and out of these around 75 percent agree that the alerts system helps reduce the time it takes to respond to a disaster event. Besides this, we find that the introduction of GDACS alerts is the system change that has generated the most significant increase in user activity in terms of number of users and comments (columns 1-3).

**Table 2:** Baseline results.

	(1)	(2)	(3)	(4)
	Timespan	Unique user count	No. of inst.	No. of comments
Redesign of VO (= 1)	1.342***	-0.019	0.184	0.327
	(0.449)	(0.114)	(0.252)	(0.277)
GDACS alerts (= 1)	0.417	0.400**	0.442*	0.532**
	(0.378)	(0.177)	(0.244)	(0.257)
Relief teams (= 1)	0.520	2.220***	0.160	1.107
	(1.021)	(0.470)	(0.947)	(1.312)
Relief items (= 1)	-0.119	0.008	-0.783**	-1.492**
	(0.543)	(0.278)	(0.394)	(0.655)
Attachments (= 1)	-0.007	0.665**	0.445	0.409
	(0.653)	(0.306)	(0.557)	(0.697)
No. of deaths (log)	0.124***	0.109***	0.220***	0.314***
	(0.032)	(0.017)	(0.022)	(0.028)
GNI per capita (log)	0.020	0.049	0.068	0.112
	(0.087)	(0.034)	(0.066)	(0.076)
Democratization	-0.007	0.011	0.023*	0.043***
	(0.019)	(0.008)	(0.013)	(0.016)
Flood	-1.279***	-0.219***	-0.454***	-0.322*
	(0.184)	(0.074)	(0.143)	(0.184)
Storm/typhoons/cyclones	-1.326***	-0.259***	-0.503***	-0.227
	(0.179)	(0.084)	(0.143)	(0.174)
Constant	0.157	2.646***	0.277	0.608
	(0.629)	(0.237)	(0.454)	(0.537)
Region dummies	Yes	Yes	Yes	Yes
Year dummies	Yes	Yes	Yes	Yes
Observations	160	215	215	215

Robust standard errors in parentheses.

*** p<0.01.

** p<0.05.

* p<0.1.

All explanatory variables (row 1-5) are dummies that take the value 1 if a disaster took place after the specific technical update was implemented. The reference disaster type is earthquake. Landslide, fire, explosion, building collapse, chemical accident, and oil-spill also included as dummies, but not reported.

The relief teams and the attachment features both seem to have generated a hike in the number of unique users (column 1). Also, we do not observe an expected decrease in the number of comments in column 3 following the introduction of these features which allow easier information sharing. In other words, although users are now able to share information on for instance relief team deployment by clicking a button instead of writing a comment, they are still active in the debate sections. In contrast, the relief item feature did not increase the number of unique users contributing to a given disaster event (column 1), but did, in line with expectations, decrease the number of comments concerning information related to relief items (column 3).

Looking at the control variables, we find that disasters with more fatalities generate more comments and hence, information sharing. The same goes for disasters that happen in countries with a higher per capita GNI, although this relationship varies across information types. The level of democracy—as measured by the Polity IV index—does not seem to have any stand-alone effect on the amount of information shared. Interestingly, user activity is independent of the region in which the event happened (results not reported). Finally, we find that floods and storms/typhoons/cyclones generate less user activity compared to the earthquakes (i.e. the left out category). This is not surprising as earthquakes were initially the event type the system was designed to address.

We now change the focus to look at outcomes related to the actual amount of information of different types shared in the system. Since we have categorized all the comments in the database according to the type of information they contain prior to the introduction of specific features, we are able to investigate how different pieces of information types are affected by the system updates. We focus on four specific features: relief teams, relief items, map feeds, and attachments. Results are presented in Table 3.

**Table 3:** The effect of features on types of information shared.

	(1)	(2)	(3)	(4)
	Relief teams	Maps	Attachments	Relief items
Relief teams feature	2.271***			
	(0.600)			
Maps feature		3.170***		
		(0.685)		
Attachment feature			0.651	
			(0.562)	
Relief items feature				-1.438***
				(0.453)
No. of deaths (log)	0.347***	0.328***	0.262***	0.250***
	(0.027)	(0.042)	(0.029)	(0.025)
GNI per capita (log)	0.160*	0.278**	0.140*	0.043
	(0.085)	(0.116)	(0.076)	(0.055)
Democratization	0.011	0.012	0.022	0.018
	(0.016)	(0.018)	(0.015)	(0.013)
Constant	-0.915	-2.627***	-1.643***	-0.681
	(0.606)	(0.816)	(0.591)	(0.421)
Disaster dummies	YES	YES	YES	YES
Region dummies	YES	YES	YES	YES
Year dummies	YES	YES	YES	YES
Observations	215	215	215	215

Robust standard errors in parentheses.

*** p<0.01.

** p<0.05.

* p<0.1.

All dependent variables are in logarithms. All explanatory variables (rows 1-4) are dummies that take the value 1 if a disaster took place after the specific technical update was implemented.

We find that two out of the four system updates have resulted in statistically significant increases in information sharing. Controlling for a number of potential observable confounders (i.e., the type and severity of the disaster as well as the circumstances under which they take place) the introduction of the relief teams feature, the maps feature and attachments feature are all positively correlated with the number of comments sparked, respectively. In fact, the introduction of the relief teams feature has more than tripled the amount of information pieces related to relief teams (column 1). The number of maps shared in the system has likewise more than quadrupled due to the introduction of the maps feed feature (column 2). The negative sign on the relief items feature should be read with caution; only 16 out of 215 disasters contain any sort of information about relief items (column 4). This is consistent with the user survey were substantially fewer users indicate that they use the relief team feature compared to the other features.

Finally, we zoom in on the relief teams feature to investigate whether highly specific sub-categories of information are affected by the system updates. Table 4 disaggregates the overall effect of user activity related to information sharing about relief teams in column 5 of Table 2. We find a clear positive effect of this technological improvement on the amount of information shared: from a doubling of team statements regarding mission completed to a tripling of statements regarding teams deployed. Only changes concerning information about team stand-by is not statistically significant at the 5 percent level. We find the strongest effect for team deployment. This is in line with the user survey where more than 70 percent of the users state that they often use the relief teams feature to get an overview of when teams are deployed. This compares to 33 percent that often use the feature to register teams for deployment

**Table 4:** The effect of the relief teams feature on sub-categories of information shared.

	(1)	(2)	(3)	(4)	(5)	(6)
	Stand-Down	Monitoring	Standby	Mobilizing	Deployed	Completed
Relief teams feature	1.613***	1.339**	0.669	1.520**	1.914**	1.064**
	(0.375)	(0.600)	(0.453)	(0.596)	(0.853)	(0.509)
No. of deaths (log)	0.176***	0.155***	0.189***	0.183***	0.305***	0.223***
	(0.027)	(0.023)	(0.019)	(0.028)	(0.030)	(0.027)
GNI per capita (log)	0.102	0.039	0.090*	0.109*	0.176**	0.105*
	(0.064)	(0.062)	(0.048)	(0.058)	(0.078)	(0.057)
Democratization	-0.009	-0.001	0.002	0.004	0.023	-0.004
	(0.012)	(0.014)	(0.011)	(0.011)	(0.016)	(0.010)
Constant	-0.793	-0.714	-0.472	-1.507***	-1.508**	-1.404***
	(0.514)	(0.453)	(0.351)	(0.437)	(0.604)	(0.416)
Disaster dummies	YES	YES	YES	YES	YES	YES
Region dummies	YES	YES	YES	YES	YES	YES
Year dummies	YES	YES	YES	YES	YES	YES
Observations	215	215	215	215	215	215

Robust standard errors in parentheses.

*** p<0.01.

** p<0.05.

* p<0.1.

All dependent variables are in logarithms. The explanatory variable (row 1) is a dummy that take the value 1 if a disaster took place after the relief teams feature was introduced.

To sum up the results, at the most general level we found that certain changes (including the GDACS alerts, relief team and relief item feature) increase information sharing activities in terms of the number of users and number of comments. Isolating user activities related to the sharing of specific types of information through categorization of user comments, we generally find that introduction of system features that enable easier submission and sorting of a particular type of information leads to more of that information type being shared. Specifically, we found that the relief team feature and the maps feature generated more user activity related to these features. Regarding the sub-categories of the relief team feature we observe a substantial effect from updates, for instance, to user activity related to team deployments. Taken together these findings suggest that advances and access to new technologies have played a larger role in populating and progressing the coordination efforts in the immediate time after the disaster.

## Qualitative Results

The quantitative analysis in the previous section builds on aggregation of user activity to the disaster level. Data aggregation in this instance is performed with the purpose of making summaries of data and for statistical analysis of the external factors for behavioral intent. Moreover, by aggregating the data to the disaster level we ensure that the external factors investigated cannot be influenced by the individual user. Yet, aggregation may conceal underlying details that are only observable at the more disaggregated level. In this section, we therefore look at the qualitative evidence for user perceptions along the lines of the TAM model. The TAM suggests that users are more likely to accept and use the system if they believe the changes will make the technology easier to use, and if they foresee the improved possibilities for information sharing in the system (i.e. usefulness) [20].

An important aspect of user’s perception of the ease of use is the level of user friendliness. Changes to the technical architecture, functionality (features), and graphical user interface are generally intended to enhance user interaction and the overall system performance. Regarding system architecture, the semi-structured interviews revealed that slow access (particularly with uploads and downloads of attachments) remains to be an issue particularly in remote locations with slow internet connectivity, indicating that the interviewees had not observed a noticeable difference throughout their use of the system. However, according to the user survey, more than two-thirds of the active users state that they are satisfied with the loading time for webpages as well as the upload/download speed for attachments.

Furthermore, to be useful in a disaster, inter-organizational information sharing technologies must work in routine use and be compatible with other systems. Thus, technological coordination tools that are not compatible with an institution’s working language, for instance, will not be used by that particular stakeholder in an actual disaster event. This is also a key message from the user survey where 80 percent of the active users state that the introduction of the multilingual interface made it easier to both find and/or contribute information.

From the data collected on user satisfaction with the technical changes and graphical user interface (GUI), respondents confirmed in the semi-structured interviews that the Virtual OSOCC has seen many changes over the years and that these changes have made the system easier to use. To quote one of them “… over the last few years, Virtual OSOCC has made major steps in terms of user friendliness, particularly the possibility to keep the overview of the information provided in Virtual OSOCC.” However one of the respondent states “…It did take me time to navigate and learn. We do get a lot of operators saying they struggle to find a way around it”. Moreover, respondents during the interviews expressed that by using the system more often, their ability to understand the technical nuances increased over time. At a more general level, 57 percent of the active users that participated in the online user survey state that they are satisfied with the GUI of the Virtual OSOCC. Table 5 lists a few basic statistics about the use and perception of Virtual OSOCC among the survey participants.

**Table 5:** Results from user survey.

	Share of respondents	Std. Dev.
When do you access the VO?		
Between disaster events	0.025	0.156
Both during and between disaster events	0.739	0.439
During disaster events	0.198	0.399
Never	0.037	0.19
What is the main purpose of the VO?		
Information sharing	0.66	0.474
Information gathering	0.198	0.399
Exchange of resources	0.089	0.284
Which group of stakeholders benefits the most from the VO?		
Governments of affected countries	0.11	0.312
Local NGOs	0.026	0.159
IGOs	0.097	0.296
International NGOs	0.161	0.368
States/organisations that plan to assist	0.529	0.499
Other	0.078	0.268

Observations: 1,044

According to the TAM model another relevant aspect to assess is how users perceive the overall benefits of using technology (i.e. system usefulness). In the quantitative analysis we found evidence to suggest that supporting technical advances has increased overall user activity as well as topic-specific information sharing. Answers by active users to the online user survey confirm these findings. According to the survey, 48 percent of the respondents state that the introduction of the relief team feature helped improve information sharing in relief coordination. Regarding the maps feature, the number is 61 percent. The least popular feature introduced to the system is the relief items feature. These findings correspond well with the average behavioral effect found in the quantitative analysis, supporting the presumption that users’ perception of the usefulness of the system is important for user uptake and actual observed use. Likewise, to the question “During an event, what is the most useful feature for sharing information?” users stated situation reports, comments and maps.

Usefulness is further determined by the relevance of the information actually shared. While the respondents mention that it is easy to find situation reports and other necessary information for disaster relief, there may also be over posting of irrelevant information. A common thread running in the interviews among the majority of the respondents concerns the type of information posted by users during a disaster. As one of the respondents notes, “What I am disappointed sometimes is with what the other information some users are putting there. You have hundreds of inputs that look of text messages or tweets because people have a tendency to mix communication channels. Often has too much quantity and very little information”. Most of the respondents echo this view that more moderation is required for all the information posted by users.

Furthermore, the relevance of the information shared also depends on the type of users actively participating and contributing to the Virtual OSOCC. As indicated previously, large scale integration across an entire network of actors is difficult to achieve as disaster response involves heterogeneous organizations and agents with a wide range of different characteristics, capabilities and capacity to collect and share information. The semi-structured interviews revealed that many responders believe that the Virtual OSOCC is predominantly a tool for select international organizations; a view that is also reflected in the user survey, where more than half of the respondents report that assisting countries and organizations are the ones who benefit most from using the Virtual OSOCC, whereas local governments of affected countries should be using the system more.

Finally, the system was initially developed to help support information sharing in disaster relief situations. This view is shared by the majority of respondents in the semi-structured interviews. Particularly, 66 percent of the users believe that the primary purpose of the Virtual OSOCC is information sharing, while the second most important purpose is information gathering (20 percent). Hence, only a fairly small share of the users thinks that the Virtual OSOCC is meant to be supporting other coordination activities such as collaboration and cooperation.

## Discussion and Conclusion

Our analysis showed that changes in the IT based platform of the Virtual OSOCC did indeed facilitate easier use of the system by users, which, subsequently led to an increase in the amount of information that was shared. However, when observing this increase we must also consider three additional elements which are relevant to disaster relief coordination: With whom is all the information shared (and with whom should it be shared)? Is the extra information necessarily relevant for other users or does it merely add to the complexity? Does the increase in information sharing lead to better coordination at a broader level? In this final section, will shall address each question in turn.

### Nature of Users

While the Virtual OSOCC is widely acclaimed in the disaster relief community, it is important to keep in mind the nature or the users. Most of the respondents to our survey expressed that it is predominantly a tool used by select international organisations and not used by governments of the affected countries. One of the respondents stated that most of the local governments have their own ways of dealing with coordination and that this system is therefore catering to a select international group of people such as the UN. While disasters involve a broad sweep of other stakeholders, they are not all using the system. This could be due to a host of reasons that needs to be explored in further research. A specific group of stakeholders that is under-represented is the national/local governments of affected countries. Some of the reasons may be attributed to non-availability of the system in the local language; presence of another system for disaster relief at the country level or even an ad-hoc system that may be created catering to all stakeholders at the local level. Further, there could also be the need for capacity building to use technology and enhance knowledge of the system and thereby increased engagement of all relevant stakeholders.

### Complexity and relevance

The study on the Virtual OSOCC is limited to the users of the system as a tool for information sharing, which can be seen as one of the most basic levels of coordination. Information technologies are argued to increase the efficiency of information exchange and hence the amount of information shared between the partners involved (i.e. an effect observed on the intensive margin). Moreover, information technologies are also likely to facilitate an increase in the number of organizations and institutions involved in information sharing—i.e. expansion of information sharing on the extensive margin. Inadequate information during relief can hamper relief efficiency and thereby affect decision making. Irrelevant information adds to the cost and time of information analysis and processing. Therefore, it is difficult to assess the net impact of increased information sharing on overall coordination in disaster relief. What is crucial during disasters is the need for *relevant* information. From our study, increased information sharing is not necessarily a proof of an increase in relevant information. The increased complexity may counteract the potential efficiency gains associated with coordination technology in the first place. However, the challenges associated with the increased complexity can be minimized or even overcome by increased moderation efforts along with continuous improvements to the technology at hand.

From our statistical analysis, it is evident that some of the technical improvements that have had the largest effect on the amount of information shared, are precisely improvements that seek to organize information and make it both easier to submit and access relevant information. For instance the introduction of the relief teams feature, which allows users to submit and browse information regarding the status of each others’ relief teams in one place without cluttering the general message board with comments had a large positive effect on the amount information shared. In our interpretation, this effect arises from the fact that the feature helps sorting and organizing information, thus reducing complexity. Easier access to relevant information was also one of the main suggestions for improvemnents from users who particitpated in our survey, so the focus for future improvements should perhaps be mechanisms that sort information either automatically, user driven, or by moderation.

### Information sharing and coordination

In the definition of coordination presented in the earlier sections of the paper interdependencies play a crucial role in coordination. As no single actor may be able to carry out all the necessary functions for efficient and quick disaster relief, there is also a need for different stakeholders to communicate and collaborate. These two elements, along with information sharing, are the basis for complex disaster relief coordination, and work together to enhance efficiency in the field. [9]. Although the main focus of the Virtual OSOCC is information sharing, there is potential that it may work as a facilitator for the other levels of coordination. There are even small signs that the type of coordination that takes place on the Virtual OSOCC is starting to go beyond information sharing. Prior to the 2015 dual earthquake in Nepal, a feature was introduced which allowed users to reply to specific comments creating a more interactive environment, which was also accompanied by a great increase in the number of comments.

That the Virtual OSOCC serves mainly as facilitator for collaboration through information sharing rather than an actual platform for collaboration is no coincidence. A lot of research (i.e. [24]; [8]; [25]) has shown that coordination processes are more or less re-invented after every disaster. Standardisation of all processes and systems is not necessarily optimal as there are immense complexities in the wake of mega disasters and that these complexities may be very contextual. However, this reinvention not only takes time and resources—it also increases pressure for relevant information given the complexity of disasters.

In summary, the empirical analysis showed that changes to the Virtual OSOCC has facilitated increased information sharing. However, as more information from more sources becomes available, the process of analyzing and utilizing the information becomes more challenging. The focus for future developments should therefore be on more flexible and dynamic coordination mechanisms that can be adapted and tailor made to different situations [26], including mechanisms to sort and navigate the information being shared. The Virtual OSOCC is unlikely to develop into a system through which all disaster relief coordination is channeled. However, since there is an ever increasing need to address complexity in a coordinated fashion, online coordination systems such as the Virtual OSOCC are likely to play even more important roles in the future. Especially, if additional key stakeholders, such as national and local governments, are increasingly involved in the coordination process.

## Supporting Information

S1 Table Category Descriptions and Numbers. (PDF)

S1 File 100_disaster_level_output. STATA do-file that generates output tables.
(DO) Click here for additional data file.
