# Supplementary material for: Technology and Information Sharing in Disaster Relief
Source: PLoS One. 2016 Sep 1;11(9):e0161783. doi: 10.1371/journal.pone.0161783 (PMC5008756; doi:10.1371/journal.pone.0161783)
Supplement: S1 Table — (PDF) [file pone.0161783.s001.pdf]

Comments categories		Number of comments	Share of comments (%)
**1.**	**Situation Reports:** General collected information (overview) about the event (location, number of people affected, magnitude, logistics). Often by UNOCHA or state officials but not always. (Links and Attachments mark separately)		
	**S1. UNOCHA or any other UN agencies.**	1,811	15.69
	**S2. Other (Other IGO, NGO or domestic government agencies)**	1,245	10.78
**2.**	**Team Features:** Comments containing information about the status of teams.		
	**T1. Stand-Down:** No activity or involvement	102	0.88
	**T2. Monitoring:** Monitoring the situation	1,069	9.26
	**T3. Standby:** Available to deploy when needed.	513	4.44
	**T4. Mobilizing:** Mobilizing to deploy.	54	0.47
	**T5. Deployed:** On route or on mission.	1,539	13.33
	**T6. Mission Completed:** Mission is finished.	183	1.59
**3.**	**Items:** Comments containing information about the status of items (non-human resources, e.g. tents, blankets, food, money, etc.)		
	**I1. Considered (standby):** Items being considered or that are available to be sent if needed.	117	1.01
	**I2. Dispatching:** Items that have been sent.	425	3.68
	**I3. Delivered:** Items that have been delivered.	206	1.78
	**I4. Canceled:** Items that will no longer be sent.	2	0.02
**4.**	**Needs/Requests:** Comments containing a request for resources, either from the affected state or the relief workers (i.e. VO users).		
	**R1. Resource:** A request for teams, equipment, funds, etc. usually by the affected state but also from relief workers.	289	2.50
	**R2. Information**: A request for information, usually by the relief workers but can also be from the affected states. This also includes answers to enquires.	455	3.94
	**R**3. **Replies** to requests for resources or information.	406	3.53
**5.**	**Maps:** Comments containing a link or attachment to a map.	73	0.63
**6.**	**Attachments:** Comments referring to attachments.	2,346	20.32
7.	**Links:** Anytime a hyperlink is included in a comment. Twitter # also	625	5.41
**8.**	**Field Coordination:** Comments containing information about meetings or field stations. This also includes comments with contact information for responders working in the field.	1,906	16,51
**9.**	**Results:** Comments containing information on outcomes or accomplishments in the field. (This also includes Updates and or general status).	845	7.32
10.	**Media:** Comments containing summaries or re-posts of media reports or news articles. Includes social media.		
	**M1. UNOCHA** posting information derived or directly from news or social media	293	2.54
	**M2. Other entities** (Other IGO, NGO or domestic government agencies) posting information derived or directly from news or social media.	522	4.52
**Total number of comments**		11,544	100
